# Editorial: Industrial and health applications of lactic acid bacteria and their metabolites, volume II

**DOI:** 10.3389/fmicb.2023.1242253

**Published:** 2023-07-04

**Authors:** Paloma López, Giuseppe Spano

**Affiliations:** ^1^Departamento de Biotecnología Microbiana y de Plantas, Centro de Investigaciones Biológicas Margarita Salas, CSIC, Madrid, Spain; ^2^Department of Agriculture Food Natural Science Engineering, University of Foggia, Foggia, Italy

**Keywords:** Lactic Acid Bacteria, probiotic, postbiotic, bacteriocin, exopolysaccharides, antioxidant activity, anti-gout activity

Lactic acid bacteria (LAB) are a heterogeneous group of species, which synthesize lactic acid as the major product of sugar fermentations. LAB are an industrially important group of microorganisms used throughout the world for a large variety of food fermentations, such as those of dairy, wine, bread, and vegetables. The European Food Safety Agency (EFSA) has introduced a system for a premarket safety assessment of selected taxonomic groups of microorganisms leading to a “Qualified Presumption of Safety” (QPS), the European equivalent of the Generally Recognized As Safe (GRAS) status. Several species of food associated LAB have obtained a QPS status. The adaptability of LAB to fermentation processes, their biosynthetic capacity and metabolic versatility, are some of the principal features that facilitate the application of LAB as microbial starters for producing, releasing and/or increasing specific beneficial compounds in fermented food. In addition, LAB produce compounds related to the food safety which just prevent the growth of pathogens (e.g., antimicrobial peptides) or conversely generating compounds which can cause serious health problems in humans (e.g., biogenic amines). Therefore, characterization and usage of new bacteriocins and antifungal compounds for food preservation by LAB are subjects of current studies in the field. LAB are also natural members of the human gastrointestinal microbiota and several strains are considered beneficial to the host and have been selected for probiotic applications. Likewise, there are a number of metabolites produced by these organisms, such as vitamins, polyphenols and gamma-aminobutyric acid (postbiotic and/or antioxidants) as well as certain polysaccharides (prebiotics and postbiotics) whose functions, among others, are to enhance the development of a microbiota that is beneficial to the human and animal gastrointestinal tract, as well as possibly possessing an immunomodulating effect. These aspects constitute an important field of research that could lead to the production of fermented functional foods (and beverages) which benefit human health.

In this context, the special issue received several contributions on the health applications of lactic acid bacteria (LAB) and their metabolites. The beneficial effect of LAB on two human trials have been reported in this special issue. First, Mozota et al. studied at the metataxonomic level the influence of *Ligilactobacillus salivarius* CECT administration during a period of 4 months in the functional, nutritional, and immunological status of elderly people living in a nursing home greatly affected by the COVID-19 pandemic. The obtained results indicated that the LAB colonized temporally the intestinal tract of the treated elderly improving the above mentioned status without altering the structure of their nasal and fecal bacteriomes at the genus level. Second, Rodríguez J. M. et al. explored the usage of LAB for alleviation of gout symptoms. The authors analyzed the capability of 13 *Ligilactobacillus salivarius* strains to degrade purines. Among them, *Ligilactobacillus salivarius CECT* 3063 showed the best ability to consume purines with no significant production of uric acid. Thus, this LAB was investigated in a randomized pilot trial on hyperuremic patients. The results showed, among other beneficial effects, a significant reduction of the gout episodies, and in the use of drugs for hyperuremic treatments.

Also, in this special issue LAB have been tested using a rat model, Jeong et al. evaluated the combination of different commercial probiotic mix (including 4 lactobacilli as well as strains belonging to the *Streptococcus thermophilus* (1) and *Bifidobacterium animalis* (1) species) on the reduction of loperamide-induced constipation. The results indicated that the probiotic mix reduce the constipation symtoms by improving the intestinal microbiota through an alteration of the levels of serotonin, mucin and short-chain fatty acids.

Many LAB have probiotic properties and they contribute to improve the properties of food during fermentation. In this context, Rodríguez J. et al. investigated the behavior of *Lactococcus lactis* LA1, *Lactococcus cremoris* LA10 and *Lactiplantibacillus plantarum* LA30 as a consortium in a fermented dairy product, performing the study by means of metabolic analysis correlated with genomic analysis.

Extracellular vesicles (EVs) present in milk or produced by probiotic bacteria have beneficial health properties. Thus, Perez Martínez et al. has investigated the protein content of EV produced by *Lacteicaseibacillus paracasei* produced in fermented dairy products.

Some compounds produced by LAB, such as exopolysaccharides (EPS) and vitamins, can act as postbiotics and immunomodulators with beneficial effects for health when produced by the bacteria *in situ* during elaboration of fermented foods. Thus, Díez-Ozaeta et al. isolated and characterized riboflavin (vitamin B_2_)-overproducing *Weissella cibaria* spontaneous mutants obtained by treatment of dextran (a EPS)- and riboflavin-producing parental strains isolated from rye sourdoughs with roseoflavin. Also, the authors proposed an *in silico* and *in vitro* procedure to detect riboflavin-overproducing mutants prior their isolation. In addition, to understand how to improve EPS production, Besrour-Ouam et al. performed a proteomic analysis of the role of the dextran production in the adaptation of the bacteria to temperature changes using as a model system *Leuconostoc lactis* AV1n isolated from avocado. A review article was presented by Pérez-Alvarado et al., in which on the basis of current knowledge the authors proposed an overview of the postbiotic-like relevance of LAB and yeasts from sourdough in breadmaking.

Hu et al. explored the antioxidant potential and probiotic properties of 15 LAB isolated from “Jiangshui” and pickled foods. Among them, two *Lactobacillus fermentans* strains (J2-5, and J2-9) shown to be the best candidates for potential future application in functional food design and healthcare.

LAB use many mechanisms for adaptation to changes in the environment and Acciarri et al. chose *Enterococcus faecalis*, a commensal bacterium of the digestive tract, to study and interrelate, its two potassium transporters with the bacterial response to alkaline and hyperosmotic stresses. High adhesion capability of probiotic bacteria could improve their beneficial effect in the gut, and Racioppo et al. has shown that ultrasound pretreatment of *Lactiplantibacillus plantarum* (but not of bifidobacteria) enhance the LAB capability to form biofilm.

In several cases, the health application of LAB deals with the antimicrobial activities of molecules produced by the tested bacteria. Vitale et al. assessed the antibiofilm activity of cell-free supernatant (CFS) of *Limosilactobacillus reuteri* DSM 17938 cultures on pathogenic microorganisms (*Escherichia coli* ATCC 25922, *Pseudomonas aeruginosa* ATCC 27853, *Staphylococcus aureus* ATCC 29213, *Streptococcus mutants* UA 159 and *Fusobacterium nucleatum* ATCC 25586 and carried out an NMR-based metabolomic exploration of the target matrix. The results of this study revealed a promising potential of the CFS and the subfractions of *Limosilactobacillus reuteri* DSM 17938 for eradication of biofilm in nosocomial and oral infections. In addition, Vanitha et al. investigated the activity of 20 LAB isolated from Haria, (a fermented rice beverage) against the dermatophyte fungus, *Trichophyton tonsurans*. Among these, the *Lactiplantibacillus plantarum* MYSN7 strain showed probiotic properties, and its CFS showed the best antifungal activity. Thus, this LAB could have a potential use as an antidermatophytic formulation. Bengoa et al. proposed the exploitation of exopolysaccharide-producing *Lacticaseibacillus paracasei* strains isolated from kefir for the production of fermented milk with antagonistic activity against intestinal pathogens. Chen et al. using cold-smoked salmon as model system, analyzed the effect of nisin on survival of three *Listeria monocytogenes* serotypes during storage. The results showed variations within *L. monocytogenes* depending on serotype, nisin concentration and temperature of storage. In addition, Nie et al. performed transcriptomic and proteomic analyses to get insight into the mechanisms involved in the enhancement of plantaricin production by *Lactiplantibacillus paraplantarum* RX-8 as a response to exposure of the BAL to the yeast *Wickerhamomyces anomalus* Y-5 by co-culture. The results showed an increase of the expression of the *L. paraplantarum plnABCDEF* cluster in the presence of the fungus as well as activation of various metabolic pathways that could provide energy for the plantaricin synthesis. Furthermore, Cebrian et al. reviewed the possible therapeutic use of AS-48, a head-to-tail cyclized cationic bacteriocin, produced by *Enterococcus faecalis*.

Finally, as an application of industrial interest, Sharma et al. reviewed the current knowledge on mechanisms of spoilage during storage of chicken and seafood products and methods for their preservation. In view of the state of the art, it is proposed the combination of LAB with essential oils as green preservative solutions for these types of food products. However, the authors state that the incorporation of these anti-spoilage agents requires assessment of their potential to alter food properties and of their possible risks.

## Author contributions

GS wrote the draft of the editorial. PL corrected the draft and generate the final version. All authors contributed to the article and approved the submitted version.

